# Is binge eating associated with poor weight loss outcomes in people with a high body weight? A systematic review with meta-analyses

**DOI:** 10.1007/s40519-023-01613-9

**Published:** 2023-10-27

**Authors:** Isabella Lobo, Felipe Q. da Luz, Phillipa Hay, Tamiris L. Gaeta, Paula Costa Teixeira, Táki Athanássios Cordás, Amanda Sainsbury, Zubeyir Salis

**Affiliations:** 1 Faculty of Medicine, Institute of Psychiatry, Eating Disorders Program (AMBULIM), University of São Paulo, São Paulo, SP Brazil; 2 School of Human Sciences, The University of Western Australia, Perth, WA Australia; 3 Faculty of Medicine, School of Public Health, Centre for Big Data Research in Health, University of New South Wales, Kensington, NSW Australia; 4 School of Medicine, Translational Health Research Institute, Western Sydney University, Sydney, NSW Australia; 5 Division of Rheumatology, Geneva University Hospital and Faculty of Medicine, University of Geneva, Geneva, Switzerland

**Keywords:** Obesity, Binge eating, Weight loss, Systematic review, Meta-analysis

## Abstract

**Objectives:**

This systematic review aimed to compare the weight change in people with or without binge eating who underwent various weight loss treatments.

**Methods:**

We searched for studies in PubMed, American Psychological Association, and Embase from inception to January 2022. The studies selected included assessment of binge eating and body weight before and after weight loss treatment in people of any age. The meta-analyses were conducted using Comprehensive Meta-Analysis (CMA). We used Egger’s regression test, the funnel plot, and the Trim and Fill test to assess the risk of publication bias.

**Results:**

Thirty-four studies were included in the systematic review, with a total of 10.184 participants. The included studies were divided into three categories according to types of weight loss treatments, namely, (1) bariatric surgery; (2) pharmacotherapy isolated or combined with behavioral interventions; and (3) behavioral and/or nutritional interventions. The meta-analyses showed no significant difference in weight loss between people with or without binge eating engaged in weight loss treatments, with an overall effect size of − 0.117 (95% CI − 0.405 to 0.171; *P* = 0.426).

**Conclusions:**

Our findings showed no difference in weight loss in people with or without pre-treatment binge eating who received various weight loss treatments. Weight loss treatments should not be withheld on the basis that they will not be effective in people with pre-treatment binge eating, albeit their safety and longer term impacts are unclear.

*Level of evidence*: Level I, at least one properly designed randomized controlled trials; systematic reviews and meta-analyses; experimental studies.

## Introduction

Obesity is a global public health concern [1]. People with obesity are at an elevated risk of weight-related health complications, such as diabetes, cardiovascular diseases, hypertension, metabolic syndrome, and fatty liver [2, 3]. In addition, some people with obesity also experience recurrent binge eating episodes [3]. Binge eating is defined as the ingestion of an excessive amount of food in a given period, typically within 2 h while feeling a sense loss of control over food intake during the episode [4]. Previous studies showed that a significant proportion of people with obesity experience binge eating episodes [5–7]. For instance, in Latin America, 16–52% of people with obesity seeking weight loss treatments had recurrent binge eating episodes [8]. In Spain, 87% of people with binge eating disorder (BED) had obesity [9], and in Finland, 56% of women with BED had overweight or obesity [10]. Moreover, the prevalence of people with obesity and comorbid binge eating has increased 7.3-fold in South Australia from 1995 to 2015 [11].

The relationship between obesity and binge eating is complex as binge eating can be a cause and a consequence of obesity [12]. Nonetheless, people with obesity and comorbid binge eating generally seek weight loss treatments rather than therapies for eating disorders [13, 14]. For instance, a systematic review found that 30–73% of people with obesity and comorbid eating disorders sought weight loss treatments [13], and another study showed that 59% of people with BED seek treatments for problems with weight [14]. In addition, a study that investigated the prevalence of BED, bulimia nervosa (BN) and recurrent binge eating in a middle-income country found that only 42.4% of people with BED sought treatment [15]. From those, 35.3% contacted a dietitian and only 25.9% sought a mental health professional (i.e., psychologist or a psychiatrist) [15]. Furthermore, previous studies found that there is a low probability that people with obesity and comorbid eating disorders receive specialized therapies for their eating disorder [16, 17]. This means that people with obesity and comorbid recurrent binge eating are mostly accessing to weight loss treatments rather than eating disorder treatments. This can be problematic, because (1) people with obesity and comorbid binge eating are thereby not accessing therapies to address their eating disorder behaviors; and (2) recurrent binge eating may potentially hinder weight loss outcomes in obesity treatments.

Previous studies showed contrasting findings regarding whether binge eating can hinder weight loss in people with obesity [18–25]. Some studies found that binge eating hinders weight loss outcomes in people with obesity [18, 19], while other studies showed that people with or without binge eating can lose weight similarly when they receive a variety of weight loss treatments [18–23]. Studies with meta-analyses also found contrasting results regarding weight loss outcomes in people with or without binge eating. A matched-study meta-analysis showed that post-treatment weight loss was poorer in people with BED compared to those without BED (1.3 kg versus 10.5 kg, respectively) [26]. Nonetheless, this matched-study meta-analysis did not investigate all the available literature in the field as it evaluated the moderating influence of binge eating on weight loss treatments that were matched to control key background variables [26]. For instance, in that matched-study meta-analysis, studies that investigated the effects of weight loss treatments in people with BED—but did not include a sample of people without BED—were matched with a sample of people without BED from another similar study [26]. In contrast, a systematic review with meta-analysis showed that pre-surgery binge eating had minimal or no effect on weight loss outcomes after bariatric surgery [21]. However, that systematic review with meta-analysis included only studies that examined weight loss in people with or without binge eating who underwent bariatric surgery, and excluded studies with other types of weight loss treatments [21].

Overall, it is not clear whether binge eating hinders weight loss outcomes in people with overweight or obesity who received weight loss treatments that are routinely implemented in clinics and hospitals. Thus, it is important to further investigate whether binge eating is associated with poor weight loss outcomes. This investigation is needed, because—if people with overweight or obesity and comorbid binge eating have poorer weight loss outcomes compared to those without binge eating—they may potentially benefit from therapies to reduce their eating disorder psychopathology and associated binge eating prior to initiating weight loss treatments. However, if weight loss is similar in people with or without binge eating, those with obesity and comorbid binge eating can potentially benefit from treatments that address weight management and eating disorder behaviors simultaneously [27]. This systematic review aims to compare changes in body weight in people with or without pre-treatment binge eating who received varied types of weight loss treatments.

## Methods

The Preferred Reporting Items for Systematic Reviews and Meta-Analyses (PRISMA) statement, updated in 2020, provided the framework for this review [28].

### Information sources

In our systematic review we searched for eligible studies in three databases, namely, PubMed, American Psychological Association (APA), and Embase.

### Search strategy

Our systematic review with meta-analyses were conducted using the following population, intervention, control, and outcome (PICO) [29] framework. The population included people with overweight or obesity and pre-treatment binge eating. The intervention comprised various weight loss treatments. The control group consisted of people with overweight or obesity but without pre-treatment binge eating. The outcome of this study was the change in body weight after a weight loss treatment.

The search terms used in each database were described below. The following search terms were used in the PubMed database: (binge eating OR binge eating disorder* OR BED) AND (weight loss* OR weight reduction*) AND (obes* OR overweight OR BMI OR body mass index), with filters for “clinical study”, “clinical trial”, “controlled clinical trial”, “randomized clinical trial”. The following search terms were used in the APA database: (binge eating OR binge eating disorder) AND (“obesity” OR overweight) AND (weight loss OR weigh reduction). The following search terms were used in the Embase database: (binge eating disorder OR binge eating) AND (obesity OR overweight) AND (body weight loss) with the filters “controlled clinical trial” and “randomized clinical trial”. An initial search was conducted in March 17th 2020 and a second search was conducted in January 21st 2022 to update our results.

### Selection process

The publications were inserted in EndNote, where duplicates were removed. Next, the studies were included in the software Rayyan, where the authors (IL, FQdL, TG) independently screened them according to eligibility criteria, initially by reading the titles and abstracts, and next by reading the full texts. Additional studies were included by active search of the reference lists of studies that met eligibility criteria.

### Eligibility criteria

We included studies that (1) assessed binge eating prior to weight loss treatments; (2) assessed body weight at pre- and post-treatment in people with overweight or obesity that were enrolled in a weight loss treatment; and (3) reported assessments of body weight in 2 or more groups of people with different levels of binge eating (e.g., subthreshold binge eating, moderate binge eating, severe binge eating, BED). Our primary outcome measure was change in body weight from baseline to the last assessment of each study. We did not include any restrictions on treatment settings/characteristics, language, date of publication, and participants’ age or sex.

We excluded studies with the following characteristics: (1) studies with animals; (2) studies that examined correlations between binge eating and weight loss but did not compare weight change in people with or without binge eating; (3) studies that included only people with recurrent binge eating or only people without binge eating; and (4) studies that did not assess pre- and post-treatment body weight in groups of people with different levels of binge eating.

### Data collection process

We extracted the following data from eligible studies: (1) characteristics of weight loss treatments; (2) sample characteristics (e.g., levels of binge eating prior to treatment, age, sex); (3) measures used to assess binge eating; and (4) body weight at baseline, end of treatment, and follow-up assessments. Data from the eligible studies were extracted by IL and checked for accuracy by FQdL.

### Data processing and meta-analyses

To conduct the meta-analyses, we compared body weight change and standard deviations (SD) of the change in weight from baseline to the end of treatment or last follow-up assessment in groups of people with or without pre-treatment binge eating and with overweight or obesity. Seven studies identified in our systematic review were eligible for a meta-analysis but did not report changes in body weight and SD of the change in weight from baseline assessment to the end of treatment or last follow-up assessment. For these studies, we employed an imputation method delineated in the Cochrane Handbook [30] to determine the SD of change in weight from baseline to the end of treatment or last follow-up assessment. The following procedure was used to include the 7 abovementioned studies in a meta-analysis:

We used data from the studies that reported change in body weight and SD of the change in weight from the baseline assessment to the end of treatment or last follow-up assessment to calculate the mean correlation. We used the formulas below to calculate the mean correlation (first formula) and SD of change in weight (second formula):

$${\text{Corr}}_{{\text{E}}} = \frac{{{\text{SD}}_{{{\text{E}},{\text{baseline}}}}^{2} + {\text{SD}}_{{{\text{E}},{\text{final}}}}^{2} - {\text{SD}}_{{{\text{E}},{\text{change}}}}^{2} }}{{2 \times {\text{SD}}_{{{\text{E}},{\text{baseline}}}} \times {\text{SD}}_{{{\text{E}},{\text{final}}}} }}$$

$${\text{SD}}_{{{\text{E}},{\text{change}}}} = \sqrt {{\text{SD}}_{{{\text{E}},{\text{baseline}}}}^{2} + {\text{SD}}_{{{\text{E}},{\text{final}}}}^{2} - \left( {2 \times {\text{Corr}} \times {\text{SD}}_{{{\text{E}},{\text{baseline}}}} \times {\text{SD}}_{{{\text{E}},{\text{final}}}} } \right)}$$

Corr is the correlation coefficient; SD_baseline_ is the standard deviation of ‘baseline’ means; SD_final_ is the standard deviation of “final” mean; SD_change_ is the standard deviation of the change between timepoints.

To minimize risk of bias, we employed the following three methods. First, some studies compared treatment effects in groups with varying levels of binge eating, such as no binge eating; moderate binge eating; and severe binge eating. To address this, we analyzed only the most extreme comparisons (i.e., no binge eating versus severe binge eating, rather than moderate binge eating versus severe binge eating). Second, the studies had different follow-up periods, so we analyzed the last assessment reported in each study. In addition, we performed two sub-group analyses based on the period of the last follow-up assessment: (a) short term (last follow-up assessment conducted less than 12 months/52.14 weeks after treatment commencement) and (b) long term (last assessment conducted at 12 months/52.14 weeks or more after treatment commencement). Third, when studies used multiple types of weight loss treatment, such as cognitive behavior therapy and behavior weight loss therapy, we analyzed each treatment independently in our meta-analyses.

We excluded from the meta-analyses studies that did not provide data that enabled the calculation of the SD of the change in body weight from pre-treatment to the end of treatment or last follow-up assessment. Studies with the following characteristics were excluded: (1) studies that did not report sample sizes in the pre-treatment phase and at the end of treatment or last follow-up assessment and (2) studies that did not report SD or standard error (SE) or did not specify whether data were reported in SD or SE. To facilitate the analyses, we converted the measurement of time to a common unit (i.e., weeks). In addition, we converted SE into SD from all the data reported in the studies. Body weight data that was reported in lb was converted to kg. However, some studies reported body weight outcomes in other units (e.g., BMI or % weight change) that could not be converted.

### Study risk of bias assessment

The quality of the studies was assessed by two authors (IS and FQdL) using criteria adapted from a checklist for the assessment of the methodological quality of randomized and non-randomized studies of health care interventions [31]. Publications were assessed on the clarity of information provided about the hypothesis or aim, outcomes, participants characteristics, main findings, attrition rates, method of randomization, allocation concealment, validity and reliability of outcome measures, blinding of participants and assessors, sample power calculation and selective outcome bias (i.e., whether or not researchers appeared to selectively report their findings). Attrition < 30% was considered acceptable. Bias was defined as the practice of reporting completers only for interventions, where attrition was > 30%. Each publication included in this systematic review was classified under each of these criteria as ‘yes’, ‘no’, ‘unclear’ or ‘not applicable’ (e.g., measures that were applicable only to randomized controlled trials), as shown in Table 2.

### Synthesis methods and certainty assessments

The meta-analyses were conducted using Comprehensive Meta-Analysis (CMA) version 3.3.070. We calculated the mean effect size and the true effect size in 95% confidence intervals. We used CMA add-on (i.e., CMA Prediction Intervals) to calculate prediction intervals. We assumed that the studies included in the meta-analyses were heterogeneous (e.g., due to differences in participants characteristics and types of weight loss treatment) and used *Q* statistic (variance of the observed effect sizes), and *I*^2^ and Tau statistics to assess heterogeneity among studies in each meta-analysis. We conducted sensitivity analyses by removing one study at a time from each meta-analysis to investigate whether the results would change from the overall result.

### Reporting bias assessment

We used the Egger’s regression test, the funnel plot and Trim and Fill test to assess the risk of publication bias. A cumulative analysis was also conducted to assess small study effects among the studies included in each meta-analysis.

## Results

### Study selection

**Fig. 1:**
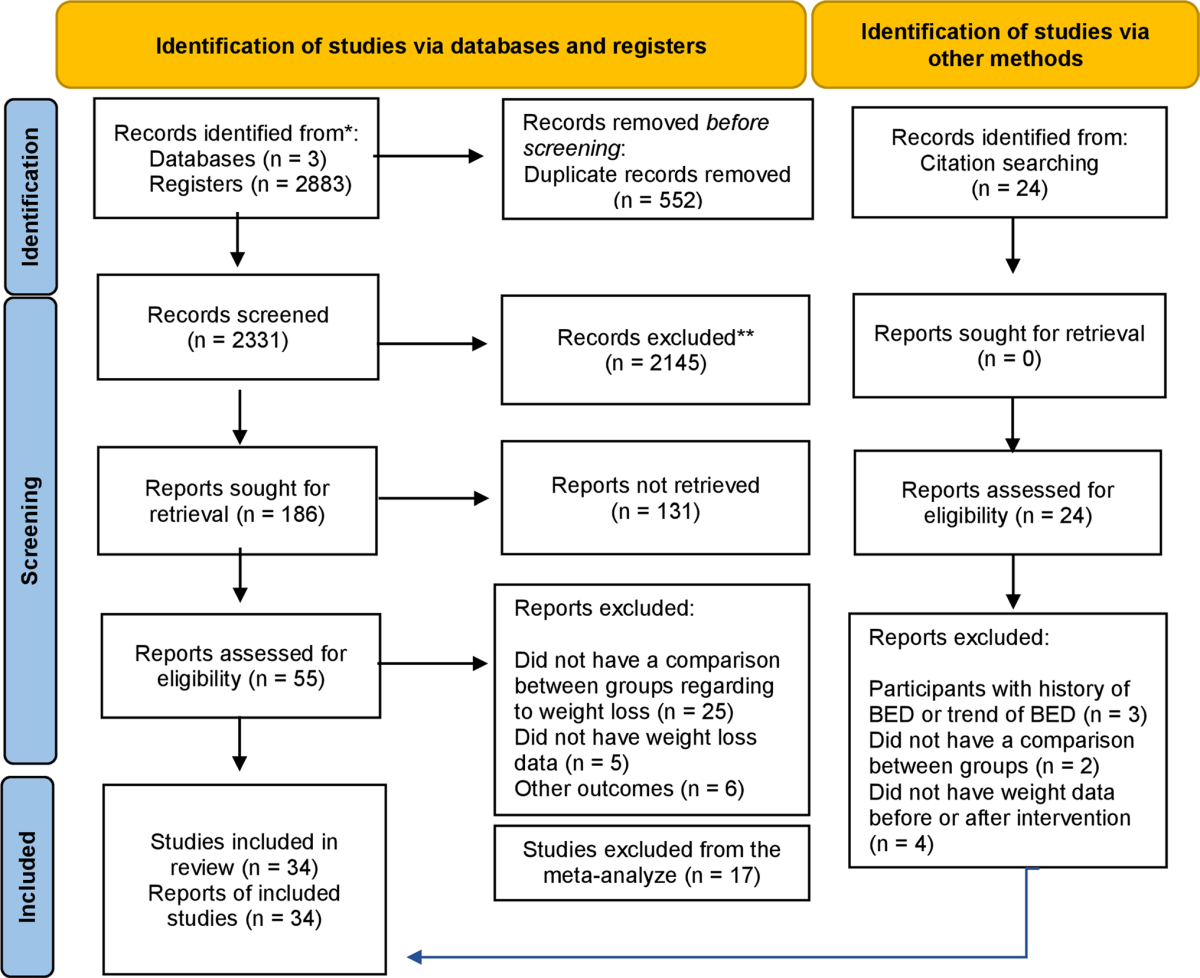
Flow chart of the systematic review search. We excluded from the meta-analyses studies that (1) did not report sample sizes in the pre-treatment phase and at the last assessment; (2) did not report SD or standard error (SE) or did not specify whether data were reported in SD or SE; and (3) studies that did not provide data that enabled the calculation of the SD of the change in body weight from pre-treatment to the end of treatment or last follow-up assessment

As shown in Fig. 1, we found 2883 publications with our search strategy. Five hundred fifty-two of these publications were duplicated, 2145 were excluded after screening titles and abstracts, and 55 were read in full. From those, 19 were included and 15 additional publications were added from reference lists of the eligible studies. In total, 34 studies were included. These studies included a total of 10.184 participants and were published from 1990 to 2021.

### Study characteristics

Study characteristics are described in Table 1. The samples size of the studies ranged from 38 to 4901 participants. Twenty-seven studies included male and female participants, and 7 studies included only female participants. Most studies (32 out of 34) included adults, 1 study included only adolescents, and 1 study included only children. The period of post-treatment assessments ranged from 10 weeks (i.e., end of treatment) to 313 weeks (i.e., extended follow-up assessment). Fourteen of the studies had a short-term follow-up period (i.e., < 12 months) and 20 studies had a long-term follow-up period (i.e., ≥ 12 months) (see Table 1).

**Table 1:** Characteristics of the included studies

Publication	Intervention	Sample size according to levels of binge eating at baseline	Binge eating assessment measure and timepoints	Mean body weight (BMI or kg or lb) in different timepoints	Weight loss comparison between groups with different levels of binge eating at baseline
Alger et al. [44]	Pharmacologic therapy using phentermine resin and dl-fenfluramine	A. participants with severe binge eating (BE) (*n* = 22) B. participants with moderate binge eating (*n* = 17) C. participants without binge eating (*n* = 16)	Binge eating scale at baseline, 3 and 6 months of treatment	Weight (lb, SD) A: Participants with severe BE Baseline: (239, 56) 3 months: (212, 53) 6 months: (204, 104) B: Participants with moderate BE Baseline: (240, 57) 3 months: (219, 58) 6 months: (214, 58) C: Participants without BE Baseline: (226, 44) 3 months: (206, 40) 6 months: (200, 41) * This paper does not explain what “S” means	There was no significant difference in percent weight loss or BMI between the groups at 3 or 6 months
Alger-Mayer et al. [32]	Roux-en-Y gastric bypass surgery *Participants attended the outpatient clinic every 3 months during the first year and annually thereafter, up to 6 years following surgery	157 preoperative adults with obesity A. Participants with score ≤ 26 on the BES (no Binge) (*n* = 120) B. Participants with score ≥ 27 on the BES (Binge eating) (* n* = 37)	Binge eating scale (BES) at baseline, every 3 months in the 1^st^ year and annually up to 6 years	Mean body weight (lb, SD) A: Participants without binge eating 3 months: (235,43) 6 months: (214, 41) 12 months: (199, 40) 24 months: (207, 44) 36 months: (216, 49) 48 months: (221, 51) 60 months: (214, 58) 72 months: (210, 52) B: Participants with binge eating 3 months: (238,48) 6 months: (210, 45) 12 months: (191, 45) 24 months: (189, 48) 36 months: (179, 35) 48 months: (188, 39) 60 months: (210, 35) 72 months: (217, 45)	No significant difference in actual weight loss, percent weight loss or excess weight loss between severe binge eaters (BES ≥ 27) and the rest of the group (BES ≤ 26) at any timepoint up to 6 years of follow-up
Balantekin et al. [54]	Family-based treatment *Family-based treatment encouraged changes in nutrition, increased physical activity, and on responsive parenting skills during 16 sessions	241 children with overweight/obesity A. Participants with low eating disorder pathology (low probability of endorsing any of the ED pathology variables) B. Participants with shape and weight concerns (high probability of reporting shape concerns and weight concerns, and a lower probability of endorsing other ED pathology) C. Participants with only loss of control (high probability of endorsing LOC eating and a lower probability of endorsing other ED pathology) D. Participants with high eating disorder pathology (high probability of endorsing all the ED pathology variables)	The children eating disorder examination at baseline and post-treatment	Participants zBMI (SD) A: Participants with low eating disorder pathology Pre-treatment: 2.10 ± 0.39 Post treatment: 1.75 ± 0.60 B: Participants with shape and weight concerns Pre-treatment: 2.24 ± 0.36 Post treatment: 1.98 ± 0.48 C: Participants with only loss of control 2.08 ± 1.74 Post treatment: 1.74 ± 0.60 D: Participants with high eating disorder pathology 2.24 ± 0.38 Post treatment: 2.08 ± 0.49	All groups significantly lost weight during family-based treatment. Children in the high eating disorder pathology and in shape and weight concerns group lost less weight in comparison with children in the low eating disorder pathology group No difference between the low eating disorder pathology and the only loss of control group
Bauer et al. [45]	1.Behavioral weight loss treatment (16 weekly 90 min group sessions) + sibutramine (10 mg/day for the first 4 weeks and 15 mg/day for the remaining 12 weeks) 2.Behavioral weight loss treatment (16 weekly 90 min group sessions) + placebo	A. Participants with obesity and subthreshold BED treated with behavioral weight loss and sibutramine (*n* = 15) B. Participants with obesity and subthreshold BED treated with behavioral weight loss and placebo (*n* = 14) C. Participants with obesity without BED treated with behavioral weight loss and sibutramine (*n* = 22) D. Participants with obesity without BED treated with behavioral weight loss and placebo (*n* = 22) *Subthreshold BED suggests a minimum of 1 binge episode per week over a period of at least 2 consecutive months	Eating disorders section of the Structured clinical interview DSM-IV ‘SKID’ (modified according to the diagnostic criteria for Subthreshold BED) and Three-factor eating questionnaire at baseline and post-treatment	Weight (kg, SD) A: Participants with subthreshold BED treated with BWL and sibutramine Baseline: 91.6 ± 8.8 End of treatment: 83.5 ± 8.5 B: Participants with subthreshold BED treated with BWL and placebo Baseline: 97.1 ± 21.5 End of treatment: 91.4 ± 20.6 C: Participants without BED treated with BWL and sibutramine Baseline: 96.1 ± 14.1 End of treatment: 88.6 ± 12.9 D: Participants without BED treated with behavioral weight loss and placebo Baseline: 104.9 ± 16.9 End of treatment: 100.2 ± 18.6	There was a moderate weight loss in all groups, with significant larger weight loss during sibutramine compared with placebo, with no difference between subthreshold BED and without BED
Ben-Porat et al. [43]	Sleeve gastrectomy	A. Participants with BED (*n* = 26) B. Participants without BED (*n* = 28)	Binge Eating Scale at baseline, 3 (M3), 6 (M6), and 12 (M12) month postoperative	(BMI, SE) A: Participants with BED Baseline BMI: (44.8, 0.9) Month 3: (35.5, 0.9) Month 6: (32.1, 0.9) Month 12: (31.2, 0.9) B: Participants without BED Baseline BMI: (45.1, 0.9) Month 3: (36.2, 0.9) Month 6: (32.7, 0.9) Month 12: (31.0, 0.9)	There was no significant difference in weight loss between participants with or without binge eating
Bishop-Gilyard et al. [55]	Participants randomized to: 1. Weekly group behavioral counseling for 16 weeks followed by bi-weekly visits for additional 8 weeks + parent separate group sessions + sibutramine 2. Weekly group behavioral counseling for 16 weeks followed by bi-weekly visits for additional 8 weeks + parent separate group sessions + placebo 41 of participants received placebo and 41 sibutramine	82 adolescents (males and females) BMI of 32 to 44 kg/m^2^ A. Participants with binge eating (*n* = 13) B. Participants with subthreshold binge eating (*n* = 7) C. Participants without binge eating (*n* = 62) *Group A and B were joint in the results to better analysis	Questionnaire on weight and eating patterns at baseline, 6 and 12 months	Group A and B: Participants with binge eating and subthreshold binge eating Baseline BMI: 38.4 (SD = 3.8) 6-month BMI: − 2.8 (SE = 0.6) 12-month BMI: − 3.3 (SE = 0.9) Group C: Participants without binge eating Baseline BMI = 37.5 (SD = 3.8) 6-month BMI: − 2.6 (SE = 0.3) 12-month BMI: − 3.1 (SE = 0.5)	There was no significantly different reduction in initial BMI between participants in the binge eating group or in the non-binge eating group at months 6 or 12
Björkman et al. [59]	1.Dietary treatment with very low energy diets 2. Dietary treatment without very low energy diet * Weight loss treatment: 12-month treatment with monthly visits	1A. Participants with BED (*n* = 120) 1B. Participants without BED (*n* = 693) 2A. Participants with severe BED (*n* = 75) 2B. Participants without BED (*n* = 244)	Questionnaire of eating and weight patterns-revised at baseline	Weight change in kg, SE Participants with binge eating: 6-month weight change: − 12.5 kg ± 0 12-month weight change: − 12.6 kg ± 1.3 Participants without BED: 6-month weight change: − 14.2 kg ± 0.4 12-month weight change: − 14.2 kg ± 0.6	Weight loss at 6 and 12 months did not differ significantly between participants with and without BED
Bocchieri-Ricciardi et al. [38]	Roux-en-Y gastric bypass (RYGBP)	A. Participants with binge eating (*n* = 24) B. Participants without binge eating (*n* = 48)	Questionnaire of eating and weight Patterns prior to surgery	(BMI, SD) A: Participants with binge eating Preoperative: 54.7 (9.4) 18-month postoperative: 34.5 (7.8) B: Participants without binge eating Preoperative: 53.6 (9.3) 18-month postoperative: 35.7 (8.0)	BMI decreased similarly for participants with pr without binge eating at 18-month post-surgery
Carbone et al. [50]	Combination of naltrexone and bupropion sustained release (NB) in addition to modified lifestyle program	People with obesity who had previously undergone at least 5 unsuccessful weight-loss programs A. Participants with BED (*n* = 23) B. Participants without BED (*n* = 20)	Binge Eating Scale Eating Disorder Examination Questionnaire 6.0 Evaluation at baseline and after 16 weeks of treatment	Weight loss after 16 weeks of treatment (BMI, SD) A: Participants with BED Baseline BMI: 39.0 ± 7.8 ΔBMI%: 8.0 ± 3.9 B: Participants without BED Baseline BMI: 43.8 ± 9.6 ΔBMI%: 7.8 ± 2.9	Weight loss was similar in participants with or without BED
Chao et al. [33]	1. Bariatric surgery: Roux-en-Y bypass or laparoscopic adjustable gastric banding 2. Lifestyle modification (no surgery): weekly group treatment sessions from week 1–20, every other week from weeks 22 to 40, and monthly sessions through week 52	A. Bariatric surgery candidates with BED (*n* = 48) B. Bariatric surgery candidates without BED (*n* = 80) C. Lifestyle modification participants with BED (*n* = 51)	Questionnaire on eating and eight patterns and eating disorder examination at 2, 6, 12 and 24 months	(Weight in kg, SE) A: Bariatric surgery candidates with BED Baseline weight 140.2 kg ± 4.2 Weight loss at month 24: − 18% ± 2.3% B: Bariatric surgery candidates without BED Baseline weight 139.3 kg ± 3.7 Weight loss at month 24: − 23.9% ± 1.6% C: Lifestyle modification participants with BED Baseline weight 125.8 kg ± 2.9 Weight loss at month 24: − 5.6% ± 1.6%	At month 24, participants with BED submitted to bariatric surgery lost significantly less weight than participants without BED submitted to bariatric surgery
Chao et al. [60]	Randomly assigned to 4 years of treatment: 1. Intensive lifestyle intervention (ILI) (*n* = 1978) *Weekly sessions for the first 6 months, 3 sessions per month for the next 6 months, and at least 1 session per month from years 2–4 2. Diabetes support and education (*n* = 2048) *3 group session each year	4.901 adults with overweight or obesity and type 2 diabetes A. “no BE”: Binge eating absent at baseline, year 1, year 2, year 3 and year 4 (*n* = 4026) B. “incident BE”: Binge eating absent at baseline but present at year 1, year 2, year 3, or year 4 C. “Fully remitted BE”: Binge eating present at baseline but absent at year 1, year 2, year 3 and year 4 D. “inconsistent BE”: Binge eating present for 2–3 years, including at baseline E. “consistent BE”: Binge eating present at ≥ 4 years, including at baseline *BE (*n* = 546)	Questionnaire on eating and weight patterns at baseline and annually (for 4 years)	A. Baseline BMI = 35.70 ± 5.77 (SD) B. Baseline BMI = 37.26 ± 6.06 (SD) Loss of initial weight at year 4: Intensive Lifestyle intervention No binge eating participants: − 4.6% ± 0.2% (SE) Incident binge eating = − 3.1% ± 0.6% (SE) Consistent binge eating lost = − 1.9% ± 1.0%. (SE) Inconsistent BE: − 3.5 ± 0.9 (SE) Fully remitted: − 4.7% ± 0.8% (SE) Diabetes support and Education No-binge eating participants:—0.9% ± 0.2% (SE) Fully remitted binge eating:—3.8% ± 0.9% (SE) Inconsistent binge eating:—3.3% ± 1.0% (SE) Incident binge eating:—0.8% ± 0.7% (SE) Consistent BE: gained 1.3% ± 1.4% (SE)	Participants in the ILI who consistently reported BE lost less weight at year 4 than participants who reported full remission of their BE after baseline or those who did not report BE at any time Participants in the DSE who reported BE at baseline (full remission of their BE after baseline or reported inconsistent BE) lost more weight than those without BE at baseline
Colles et al. [34]	Laparascopic adjustable gastric banding surgery (LAGB)	A. Participants with severe obesity and BED (*n* = 18) B. Participants with severe obesity and uncontrolled eating (*n* = 40) C. Participants with severe obesity and night eating syndrome (NES) (*n* = 22) D. Participants with severe obesity and grazing (*n* = 34) Groups A and B were combined in the results, because after surgery, few participants continued to have full criteria for BED and were too few for statistical analysis	The questionnaire on eating and weight patterns-revised at baseline and 12 months after LAGB	Group A and B (Uncontrolled eaters) BMI at baseline = 44.8 ± 6.9 (SD) BMI at 4 m = 39.2 ± 6.5 (SD) BMI at 12 m = 37.0 ± 7.1 (SD) %WL = 17.4 ± 8.2 (SD) Participants with BED 12 months after surgery: %WL 21.9% ± 11.1% Remainder of cohort (NES and grazing group) BMI at baseline = 44.1 ± 6.8 BMI at 4 m = 38.1 ± 5.6 (SD) BMI at 12 m = 34.3 ± 5.4 (SD) %WL = 22.0 ± 8.3 (SD)	Baseline BED achieved a significant similar weight loss to the remainder of the cohort at 12 months after surgery
Delinsky et al. [51]	Trevose Behavior Modification Program (TBMP) *TBMP is a lay-administered, lay-directed, self-help weight loss program. Weekly sessions	A. Participants with Binge eating (*n* = 27) B. Participants without binge eating (*n* = 134)	Eating Disorder examination-questionnaire and Questionnaire on Eating and Weight Patterns-Revised	A: Participants with Binge eating Baseline BMI: 34.31 (SD = 6.6) Weight loss at month 1: − 5.12 kg (SD = 1.27) Weight loss at month 12: − 12.45 kg (SD = 1.02) B: Participants without binge eating Baseline BMI: 34.98 (SD = 4.53) Weight loss at month 1: − 4.96 kg (SD = 1.59) Weight loss month at 12: − 18.71 kg (SD = 8.09)	No statistically significant difference in weight loss between participants with or without BED
Gladis et al. [63]	Participants randomly assigned to 48 weeks of: 1. Diet + behavioral treatment 2. Diet + behavioral treatment + aerobic training 3. Diet + behavioral treatment + strength training 4. Diet + behavioral treatment + aerobic and strength trained combined	A. Participants with obesity without overeating (*n* = 59) B. Participants with obesity and episodic overeating (*n* = 22) C. Participants with obesity and subthreshold BED (*n* = 23) D. Participants with obesity and BED (*n* = 14)	Questionnaire on eating and weight patterns at baseline, 8 weeks, 17 weeks, 24 weeks, 48 weeks and follow-up	Weight in kg, S (not specified if SD or SE) A: Participants without overeating Baseline weight = 95.3 ± 11.9 Mean weight loss: 8 weeks: 10.3 ± 2.8 17 weeks: 14.6 ± 5.0 24 weeks: 16.4 ± 5.6 48 weeks: 13.8 ± 6.5 Follow-up: 6.7 ± 6.6 B: Participants with episodic overeating Baseline weight: 91.7 ± 12.7 Mean weight loss: 8 weeks: 8.1 ± 3.5 17 weeks: 12.7 ± 5.9 24 weeks: 14.6 ± 6.7 48 weeks: 12.8 ± 8.4 Follow-up: 7.8 ± 10.9 C: Participants with subthreshold BED Baseline weight: 96.9 ± 11.5 Mean weight loss: 8 weeks: 11.2 ± 3.9 17 weeks: 15.4 ± 5.3 24 weeks: 17.2 ± 6.3 48 weeks: 14.6 ± 7.4 Follow-up: 8.8 ± 7.9 D: Participants with BED Baseline weight: 103.8 ± 22.1 Mean weight loss: 8 weeks: 12.6 ± 4.8 17 weeks: 18.7 ± 7.3 24 weeks: 23.1 ± 10.0 48 weeks: 22.7 ± 13.0 Follow-up: 15.8 ± 12.9	The BED group lost the most weight and the episodic overeaters the least. The BED group continued to maintain a greater absolute weight loss than other groups at follow-up
Green et al. [35]	Roux-en-Y gastric bypass surgery (RYGBP)	A. Participants with obesity and binge eating (*n* = 33) B. Participants with obesity without binge eating (*n* = 32)	Eating Disorders Module, Structured Clinical Interview and Questionnaire on Eating and Weight Patterns-Revised at baseline	Weight loss from pre-surgery to post surgery (lb, SD) A: Participants with Binge Eating Baseline weight: 348.3 (SD = 85.1) Post-surgery weight: 261.1 (SD = 64.7) *Weight loss of 86.1 (SD = 27.5) B: Participants without Binge eating Baseline weight: 333.1 (SD = 62.1) Post-surgery weight: 243.3 (SD = 54.7) *Weight loss of 89.7 (SD = 22.8)	The binge eating group lost less percent of excess weight than the non binge eating group
Grilo et al. [48]	1.Orlistat + Behavioral weight loss treatment (120 mg 3x/daily for 4 months) 2.Placebo + Behavioral weight loss treatment	A. Participants with obesity and BED (*n* = 40) B. Participants with obesity without BED (*n* = 39)	Structured clinical interview for DSM-IV Axis 1 (SCID-I/P) and Eating Disorder Examination interview at baseline, post treatment and 6 months of follow-up	Mean BMI (SD) 1.A Participants with BED (Orlistat) Baseline BMI: 39 (7.0) Post-treatment: 37.9 (6.9) 6 months of follow-up: 37.6 (5.7) 1.B: Participants without BED (Orlistat) Baseline BMI: 35.2 (3.3) Post-treatment: 33.6 (3.4) 6 months of follow-up: 35.3 (3.1) 2.A: Participants with BED (Placebo) Baseline BMI Baseline BMI: 37.2 (5.3) Post-treatment: 36.0 (5.0) 6 months of follow-up: 36.7 (5.3) 2.B: Participants without BED (Placebo) Baseline BMI: 37.4 (4.7) Post-treatment: 36.6 (4.5) 6 months of follow-up: 36.7 (4.0)	There was no statistical analysis comparing weight change between participants with or without binge eating
Grilo et al. [49]	1. Orlistat (120 mg three times daily, fixed dose, per previous studies) 2. Placebo (three times daily) * All participants received 4 months of behavioral weight loss treatment in individual, 60-min weekly sessions	A. Participants with obesity and BED (*n* = 40) B. Participants with obesity without BED (*n* = 39)	Eating Disorder Examination at baseline, monthly throughout treatment, at post-treatment and at the 6-month follow-up after completing treatment	(BMI, SD) A: Participants with BED Baseline BMI: 38.11 (SD = 6.20) Post-treatment (4 months): 36.86 (SD = 6.05) Follow-up of 6 months: 36.59 (SD = 5.56) B: Participants without BED Baseline BMI: 36.30 (SD = 4.16) Post-treatment: 35.44 (SD = 4.95) Follow-up: 36.08 (SD = 3.77)	There was no statistical analysis comparing weight change between participants with or without binge eating
Kops et al. [39]	Roux-en-Y gastric bypass	A. Participants with lower score in BES (*n* = 63) B. Participants with higher score in BES (*n* = 45) *Lower score (BES between 0 and 17 8.7 ± 4.6; median 9); higher score (between 18 and 40 points 27.0 ± 5.8; median 27)	Interview using the Structures clinical interview for DSM-IV Axis 1 Disorders (SCID) and Binge Eating Scale (BES) at baseline	Weight (Kg), SE A: Participants with lower score in BES Baseline: 126.7 (2.6) 3 months: 106.1 (2.5) 6 months: 94.1 (2.1) 12 months: 87.0 (2.0) 24 months: 86.1 (1.9) 36 months: 88.9 (2.2) 48 months: 90.0 (2.9) B: Participants with higher score in BES Baseline: 128.0 (2.7) 3 months: 104.0 (2.9) 6 months: 93.3 (2.9) 12 months: 84.5 (2.3) 24 months: 82.5 (2.2) 36 months: 84.9 (2.4) 48 months: 86.3 (2.3) 60 months: 87.0 (3.4)	Binge eating group lost more percentage total body weight loss than those without binge eating in months 3, 24 and 36. However, this difference was not found at other timepoints
LaPorte et al. [56]	Combination of behavior modification and partial fasting on a very low-calorie diet (VLCD) *10 weeks, for 2 h each	A. Participants with binge eating (*n* = 25) B. Participants without binge eating (*n* = 24)	Binge eating scale at baseline	A: Participants with binge eating Baseline weight: 108.1 kg ± 18.8 Weight loss by week 10: 18.72 kg B: Participants without binge eating Baseline weight: 109.0 kg ± 22.4 Weight loss by week 10: 20.23 kg	There were no significant differences between people with or without binge eating on the amount of weight lost by the end of treatment
Marcus et al. [47]	Randomly assigned to 1. Behavior modification program + fluoxetine 2. Behavior modification program + placebo	1.A. Participants without binge eating treated with fluoxetine (*n* = 13) 1.B. Participants with binge eating treated with fluoxetine (*n* = 10) 2.A. Participants without binge eating treated with placebo (*n* = 10) 2.B. Participants with binge eating treated with placebo (*n* = 12)	Binge eating scale at baseline	Weight in kg, SD 1.A: Participants without binge eating (fluoxetine) Baseline weight: 92.8 (15.9) Weight loss after treatment 52 weeks: 17.1 kg 1.B: Participants with binge eating (fluoxetine) Baseline weight: 103.9 (16.0) Weight loss after treatment (52 weeks): 8.4 kg Participants treated with placebo gained 0.6 kg in the end of the treatment *No data of the placebo + behavior modification program with or without binge eating	Binge eating status did not affect post-treatment weight change Participants without binge eating + fluoxetine lost more weight than the rest of the groups
Masheb et al. [61]	Participants were randomized to: 1.ASPIRE-phone (*n* = 128) 2. ASPIRE-group (*n* = 132) 3. MOVE!: Veterans health administration national weight management program (“usual care”); (*n* = 132) Aspire and Move treatments were held for 3 months of weekly treatment + 9 months of maintenance (biweekly sessions for 6 months and monthly sessions for 3 months)	392 Veterans with overweight or obesity and at least one obesity-related health condition A. Participants without binge eating (participants who reported “never” binge eating/*n* = 88) B. Participants with any binge eating (participants who reported “less than one time per week” binge eating/*n* = 117) C. Participants with high binge eating (participants who reported binge eating 5 or more times per week/*n* = 24) D. Participants with not high binge eating (all others participants/*n* = 163)	The binge eating behavior item from the MOVE!23 survey	Weight in kg, SD A: Participants without binge eating Baseline weight: 107.2 (18.2) Weight loss at 12 months: − 2.9 kg B: Participants with any binge eating Baseline weight: 114.1 (23.6) Weight loss at 12 months: − 1.6 kg C: Participants with high binge eating Baseline weight: 112.4 (22.6) Weight loss at 12 months: − 1.6 kg D: Participants with not high binge eating Baseline weight: 114.9 (23.5) Weight loss at 12 months: − 2.1 kg	The “no binge eating group” had significantly greater mean percent weight loss than “any binge group” by the end of the treatment The “not high binge eating group” had significantly greater mean percent weight loss, and greater reduction in weight and BMI than the “high binge group”
Nauta et al. [53]	74 women with or without BED were randomly assigned to: 1. Cognitive treatment (CT) (15 weekly sessions of 150 min each) 2. Behavioral treatment (BT) (15 weekly sessions of 150 min each)	A. Participants without binge eating submitted to behavioral treatment (*n* = 21) B. Participants with binge eating submitted to behavioral treatment (*n* = 16) C. Participants without binge eating submitted to cognitive treatment (*n* = 16) D. Participants with binge eating submitted to cognitive treatment (*n* = 21)	Eating disorder examination questionnaire at baseline, post-treatment and 6-month follow-up	Weight in kg, SD A: Participants without binge eating (BT) Baseline weight: 92.6 (SD = 9.7) Post-treatment weight: 87.9 (SD = 9.9) Follow-up weight: 89.8 (SD = 9.4) B: Participants with binge eating (BT) Baseline weight: 96.6 (SD = 16.4) Post-treatment weight: 90.4 (SD = 15.0) Follow-up weight: 94.6 (SD = 16.8) C: Participants without binge eating (CT) Baseline weight: 88.8 (SD = 11.1) Post-treatment weight: 88.3 (SD = 12.3) Follow-up weight: 89.1 (SD = 13.1) D: Participants with binge eating (CT) Baseline weight: 95.5 (SD = 15.5) Post-treatment weight: 94.2 (SD = 15.5) Follow-up weight: 95.4 (SD = 16.7)	Participants with obesity and comorbid binge eating showed a significant weight gain from post-treatment to follow-up, while participants with obesity that did not have binge eating showed no significant weight gain
Nikiforova et al. [36]	Laparoscopic sleeve gastrectomy surgery	A. Participants with binge eating (*n* = 42) B. Participants without binge eating (*n* = 258)	Eating habits, activities, and weight questionnaire periodically during the 3-year of follow-up	Weight in kg, SD A: Participants with binge eating Baseline weight: 113.76 (SD = 19.99) 1 year after surgery: 80.47 (SD = 16.49) B: Participants without binge eating Baseline weight: 117.97 (SD = 19.00) 1 year after surgery: 80.99 (SD = 14.76)	There was no significant difference in weight loss in the binge-eating group and no binge eating group 1 year after surgery
Porzelius et al. [52]	1.Standard behavioral program 2. Obese binge eating treatment (OBET) Both were 15, 90 min session groups over a 17-week period	A.1: Participants with moderate binge eating (Standard) (*n* = 8) A.2: Participants with moderate binge eating (OBET) (*n* = 10) B.1: Participants with severe binge eating (Standard) (*n* = 9) B.2: Participants with severe binge eating (OBET) (*n* = 11) C.1: Participants without binge eating (standard) (*n* = 8) C.2: Participants without binge eating (OBET) (*n* = 8)	Binge eating scale (prior to treatment, post treatment, and 12-month follow-up)	A. Participants with moderate binge eating (Standard) Pre-treatment weight: 82.5 kg (SD = 13.4) Post-treatment: 74.7 kg (SD = 14.8) Follow-up: 79.5 kg (SD = 16.9) A.2: Participants with moderate binge eating (OBET) Pre-treatment weight: 80.5 kg (SD = 12.4) Post-treatment: 79.6 kg (SD = 12.4) Follow-up: 77.8 kg (SD = 15.3) B.1: Participants with severe binge eating (Standard) Pre-treatment weight: 80.5 kg (SD = 9.9) Post-treatment: 74.4 kg (SD = 12.2) Follow-up: 77.5 kg (SD = 10.2) B2: Participants with severe binge eating (OBET) Pre-treatment weight: 86.7 kg (SD = 14.4) Post-treatment: 82.0 kg (SD = 18.9) Follow-up: 81.5 kg (SD = 21.9) C.1: Participants without binge eating (standard) Pre-treatment weight: 85.5 kg (SD = 11.5) Post-treatment: 79.8 kg (SD = 9.6) Follow-up: 86.1 kg (SD = 7.2) C.2 Participants without binge eating (OBET) Pre-treatment weight: 81.9 kg (SD = 12.2) Post-treatment: 75.7 (9.3) Follow-up: 76.1 kg (12.7)	Standard treatment: there were no significant differences in weight loss between women with no, moderate, or severe binge eating in standard treatment OBET: there were no statistical comparison between participants with or without binge eating; however, participants with severe binge eating lost significantly more weight than those with moderate binge eating
Puglisi et al. [40]	Intragastric balloon (BIB)	75 Candidates for treatment with Intragastric balloon A. Participants with binge eating (*n* = 27) B. Participants without binge eating (*n* = 48)	Eating Disorder Module of the Structures clinical interview for DSM-IV Axis 1 Disorders (ED-SCID) and Binge Scale Questionnaire (BSQ) Applied Before the treatment and after extraction of the BIB	A. Participants with Binge Eating T0: pre-implement: 122 kg (SD ± 19.1) T1: at removal of BIB (6 months after T0): 106.3 kg (SD ± 18.4) T2: 3 months after removal of BIB (9 months after T0): 104.2 kg (SD ± 19.8) B. Participants without Binge Eating T0: Pre-implement: 136 kg (SD ± 24.1) T1: at removal of BIB (6 months after T0): 115.2 kg (SD ± 23.69) T2: 3 months after removal of BIB (9 months after T0): 118.4 kg (SD ± 24.8)	Participants without binge eating showed a greater reduction of BMI in comparison with the binge eating group at 9-month post BIB There was no significant difference at other timepoints
Raymond et al. [57]	24-week very low-calorie diet program + weekly psychoeducational/behavioral weight management group meetings + consultation with a physician every other week. After 2 weeks of refeeding phase, half of the participants with BED were randomly assigned to additional 10 weeks of cognitive behavior therapy	A. Participants with obesity and BED (*n* = 63) B. Participants with obesity and subthreshold BED (*n* = 36) C. Participants with obesity without binge eating (*n* = 29)	Structured clinical interview for DSM-IV (eating disorders section only) and Eating behaviors questionnaire at baseline and 1-year follow-up	A. Participants with BED Weight loss during 24 weeks of treatment = 38.5 lb ± 18.4 (SD) Weight gain at 1y follow-up: 22.2 lb ± 16.7 (SD) B. Participants with subthreshold BED Weight loss during 24 weeks of treatment = 42.0 lb ± 17.8 (SD) Weight gain at 1y follow-up: 26.8 lb ± 12.4 (SD) C. Participants without binge eating Weight loss during 24 weeks of treatment = 30.3 lb ± 16.8 (SD) Weight gain at 1-year follow-up: 18.2 lb ± 13.3 (SD)	There were no differences among the groups in weight lost during the fast
Sallet et al. [41]	Roux-en-Y gastric bypass surgery	A. Participants with obesity without binge eating (NBE) (*n* = 43) B. Participants with obesity and subclinical binge eating (fewer than 2 episode of binge/week-SBE) (*n* = 129) C. Participants with obesity and binge eating disorder (BED) (*n* = 44)	Semi-structured psychiatric interview using the Structures clinical interview for DSM-IV Axis 1 Disorders (SCID-P) at baseline	Percent of excess BMI lost (%EBL) A: Participants without binge eating Baseline BMI: 46.1 ± 6.0 6-month follow-up: 60.1 + 18.1 1-year follow-up: 78.2 ± 21.8 2-year follow-up: 91.3 ± 18.5 3-year follow-up: 91.8 ± 17.7 B: Participants with SBE Baseline BMI: 45.9 ± 6.1 6-month follow-up: 57.6 ± 13.9 1-year follow-up: 67.6 ± 21.7 2-year follow-up: 76.1 ± 21.8 3-year follow-up: 66.1 ± 18.6 C: Participants with BED Baseline BMI:45.6 ± 5.8 6-month follow-up: 56.8 ± 14.4 1-year follow-up: 67.6 ± 17.0 2-year follow-up: 70.1 ± 19.5 3-year follow-up: 63.8 ± 17.7	No significant difference in weight loss between groups during 6 months of follow-up During 1 and 2 years of follow-up, participants with NBE showed higher %EBL than SBE and BED participants, showing a significant difference in 2 years of follow-up SBE and BED groups were not statistically different at any period of follow-up
Susmallian et al. [42]	Laparoscopic sleeve gastrectomy surgery (LSG)	A. Participants with binge eating (*n* = 42) B. Participants without binge eating (*n* = 258)	Interview conducted by two nutritionist which included a questionnaire that evaluated the type, quality, quantity, and time intervals of the patients’ eating habits	Weight in kg, SD A: Participants with binge eating Before surgery: 113.76 (SD = 19.99) 1 year after surgery: 80.47 (SD = 16.49) B: Participants without binge eating Before surgery: 117.97 (SD = 19.0) 1 year after surgery: 80.99 (SD = 14.76)	Participants without BE habits lost more of their excess weight and BMI than those with BE habits 1-year post-surgery
Telch et al. [58]	12 weeks of very low caloric diet + gradual refeeding and behavior therapy for 9 months	A. Participants with obesity and binge eating (*n* = 20) B. Participants with obesity without binge eating (*n* = 71) * The remainder met some, but not all, of the criteria for binge or not binge and were excluded from the sample	Stanford eating behavior questionnaire at baseline, at the end of the first week, months l, 3, 6, 9, 12 and 15	A: Participants with binge eating Baseline: 104.5 kg 3-month follow-up: 94.4 kg B: Participants without binge eating Baseline: 99.5 kg 3-month follow-up: 89.7 kg	There were no significant differences in weight loss in participants with or without binge eating at any timepoint
Tseng et al. [64]	Group-based behavioral weight loss program + exercise sessions *12-week program, twice per week for 4 weeks, and weekly sessions from week 5–12 of advanced exercise sessions	A. Participants with obesity and binge eating (*n* = 30) B. Participants with obesity and subthreshold binge eating (SBE) (*n* = 76) C. Participants with obesity without binge eating (*n* = 83)	The bulimic investigation test at pre and post treatment	Mean body weight loss (kg) of completers, SE A: Participants with binge eating Initial weight: 79.4 ± 16.3 Weight loss at end of treatment (12 weeks): 4.4 ± 1.2 Weight loss at 36 weeks: 4.2 ± 1.9 B: Participants with SBE Initial weight: 79.8 ± 14.4 Weight loss at end of treatment (12 weeks): 8.6 ± 0.6 Weight loss at 36 weeks: 8.4 ± 1.0 C: Participants without binge eating Initial BW: 78.8 ± 12.6 Weight loss at end of treatment (12 weeks): 6.4 ± 0.6 Weight loss at 36 weeks: 6.7 ± 0.9	No significant differences in weight loss over the course of the 12-week treatment among participants with binge eating, SBE, or without binge eating No significant differences in weight regain at follow-up (6 months after the end of treatment) among groups with different levels of binge eating
Wadden et al. [62]	26-week weight reduction program *Week 1: 1.200 kcal per day balanced deficit diet weeks 2–13: very low-calorie liquid diet; weeks 14–19, conventional foods were gradually reintroduced; weeks 20–26: subjects were instructed to consume a 1.200–1.500 kcal per day balanced deficit diet of their own choosing	A. Participants with binge eating (*n* = 29) B. Participants with episodic overeating (*n* = 26) C. Participants without binge eating (*n* = 180)	Weight and Lifestyle Inventory at baseline	A: Participants with binge eating Baseline weight: 97.8 kg (SD = 17.2) Mean weight losses: Week 5: 7.6 kg (SD = 5.1) Week 9: 13.5 kg (SD = 6.0) Week 13: 17.8 kg (SD = 7.4) Week 19:19.8 kg (SD = 9.8) Week 26: 21.5 kg (SD = 8.9) B: Participants with episodic overeating Baseline weight: 99.0 kg (SD = 14.5) Mean weight losses: Week 5: 8.9 kg (SD = 1.9) Week 9: 14.7 kg (SD = 3.4) Week 13: 18.8 kg (SD = 5.5) Week 19: 22.0 kg (SD = 7.8) Week 26: 19.4 kg (SD = 10.5) C: Participants without binge eating Baseline weight: 101.6 kg (SD = 16.6) Mean weight losses: Week 5: 8.3 kg (SD = 3.4) Week 9: 14.0 kg (SD = 3.4) Week 13: 18.9 kg (SD = 5.8) Week 19: 21.4 kg (SD = 7.0) Week 26: 21.7 kg (SD = 8.8)	No significant differences in weight losses were observed among the 3 groups at the end of the very low-calorie diet (week 13), after the refeeding period (week 19), end of the end of treatment
Wadden et al. [37]	1.Bariatric surgery: Roux-en-Y gastric bypass or adjustable gastric banding (*n* = 151) 2. Lifestyle modification: group program with weekly sessions from weeks 1 to 20, bi-weekly sessions from weeks 22 to 40, and monthly sessions through week 52 (*n* = 57)	1A. Participants with BED submitted to bariatric surgery (*n* = 51) 1B. Participants without BED submitted to bariatric surgery (*n* = 80) 2A. Participants with BED submitted to a lifestyle modification intervention (*n* = 51)	Questionnaire on eating and weight patterns (before treatment) and eating disorder examination at baseline, months 2, 6, and 12	Changes at 1 year in weight (kg, % change) 1A: Participants with BED (bariatric surgery) Baseline weight: 138.7 ± 4.0 Changes at 1 year in weight: − 29.2 kg ± 2.2 (SE) 1B: Participants without BED (bariatric surgery) Baseline weight: 139.3 kg ± 3.7 Changes at 1 year in weight: − 33.3 kg ± 1.7 (SE) 2: Participants with BED (lifestyle modification) Baseline weight: 125.8 ± 2.9 Changes at 1 year in weight: − 15.6 ± 2.0 (SE)	Weight loss of the two surgical groups (with or without BED) did not differ at months 2, 6 and 12 The presence of subjective binge episodes, whether present before surgery or at the 12-month evaluation, did not affect weight loss
Yanovski et al. [65]	Very-low-calorie diet *26-week weight loss treatment	A. Participants with binge eating (*n* = 21) B. Participants without binge eating (*n* = 17)	Questionnaire on Eating and weight patterns and Binge eating scale	A: Participants with binge eating Baseline weight: 114.3 kg ± 5.5 By the end of 26-week weight loss program: − 19.6 ± 2.5 kg B: Participants without binge eating Baseline weight: 104.5 kg ± 4.3 By the end of 26-week weight loss program: − 21.3 ± 11.4 kg	There was no significant difference in percentage of initial body weight lost between people with or without BED at any timepoint
Zwaan et al. [46]	1.Cognitive behavioral treatment + fluvoxamine 2. Cognitive behavioral treatment + placebo 3. Dietary management + fluvoxamine 4. Dietary management + placebo *No information about the design of each treatment	Women with a history of a major affective disorder and/or emotional disturbances secondary to dieting A. Participants with binge eating (*n* = 22) B. Participants without binge eating (*n* = 42)	Structured clinical interview for DSM III-R and the diagnostic survey of eating disorder	A: Participants with binge eating Baseline BMI: 35.8 kg ± 5.6 (SD) Weight change: − 6.12 kg ± 4.7 (SD) Follow-up: + 4.63 kg ± 9.9 (SD) B: Participants without binge eating Baseline BMI: 36.7 kg ± 4.7 (SD) Weight change: − 5.61 kg ± 3.7 (SD) Follow-up: + 1.55 kg ± 5.1 (SD)	Participants with and without binge eating did not differ in amount of weight loss at the end of the active treatment and at the 1-year follow-up

*BES* Binge eating scale, *SD* standard deviation, *SE* standard error, *ED* eating disorder, *BED* binge eating disorder, *BE* binge eating, *NBE* without/no binge eating, *NBED* without/no binge eating disorder, *SBE* subthreshold binge eating, *SBED* subthreshold binge eating disorder, *LOC* loss of control, *BMI* body mass index, *BWLT* behavioral weight loss treatment, *RYGBP* Roux-en-Y gastric bypass, *ILI* intensive lifestyle intervention, *TBMP* Trevose Behavior Modification Program, *BES* Binge Eating Scale, *CT* cognitive treatment, *BT* behavioral treatment, *OBET* obese binge eating treatment, *BIB* intragastric balloon, *LSG* laparoscopic sleeve gastrectomy surgery

The included studies were divided into 3 categories according to types of weight loss treatments: (1) bariatric surgery; (2) pharmacotherapy isolated or combined with behavioral interventions; and (3) behavioral and/or nutritional interventions. We found 12 studies with bariatric surgery [32–43], 7 studies with pharmacotherapy isolated or combined with behavioral interventions [44–50], and 15 studies with behavioral and/or nutritional interventions [51–65] (Table 2).

**Table 2:** Quality of the studies included in the systematic review

Publication	Hypothesis or aim and outcomes clearly described	Participants characteristics and main findings clearly described	Attrition rates (dropouts) reported and acceptable	Method of randomization	Allocation concealment	Outcome measures valid and reliable	Blind participants and assessors	Sample power calculation	Absence of selective outcome bias
Alger et al. [44]	Yes	Yes	Yes	Not applicable	Not applicable	Yes	No	Unclear	Yes
Alger-Mayer et al. [32]	Yes	Yes	Yes	Not applicable	Not applicable	Yes	No	Unclear	Yes
Balantekin et al. [54]	Yes	Yes	Yes	Not applicable	Not applicable	Yes	No	Unclear	Yes
Bauer et al. [45]	Yes	Yes	Yes	No	No	Yes	No	Yes	Yes
Ben-Porat et al. [43]	Yes	Yes	Yes	No	No	Yes	No	Unclear	Yes
Bishop-Gilyard et al. [55]	Yes	Yes	No	No	No	Yes	Yes	Unclear	Yes
Björkman et al. [59]	Yes	Yes	No	Not applicable	Not applicable	Yes	No	Unclear	Yes
Bocchieri-Ricciardi et al. [38]	Yes	Yes	Yes	Not applicable	Not applicable	Yes	No	Unclear	Yes
Carbone et al. [50]	Yes	Yes	Yes	Not applicable	Not applicable	Yes	No	Unclear	Yes
Chao et al. [33]	Yes	Yes	No	Not applicable	Not applicable	Yes	No	Unclear	Yes
Chao et al. [60]	Yes	Yes	Yes	Yes	Yes	Yes	No	Yes	Yes
Colles et al. [34]	Yes	Yes	Yes	Not applicable	Not applicable	Yes	No	Unclear	Yes
Delinsky et al. [51]	Yes	Yes	No	Not applicable	Not applicable	Yes	No	Unclear	Yes
Gladis et al. [63]	Yes	Yes	Yes	No	No	Yes	No	Unclear	Yes
Green et al. [35]	Yes	Yes	Yes	Not applicable	Not applicable	Yes	No	Unclear	Yes
Grilo et al. [48]	Yes	Yes	Yes	Yes	Yes	Yes	Yes	Yes	Yes
Grilo et al. [49]	Yes	Yes	Yes	No	Yes	Yes	Yes	Unclear	Yes
Kops et al. [39]	Yes	Yes	Yes	Not applicable	Not applicable	Yes	No	Unclear	Yes
LaPorte et al. [56]	Yes	Yes	No	Not applicable	Not applicable	Yes	No	Unclear	Yes
Marcus et al. [47]	Yes	Yes	No	Yes	Yes	Yes	No	Unclear	Yes
Masheb et al. [61]	Yes	Yes	Yes	Yes	Yes	No	No	Yes	Yes
Nauta et al. [53]	Yes	Yes	Yes	No	No	Yes	No	Unclear	Yes
Nikiforova et al. [36]	Yes	Yes	Yes	Not applicable	Not applicable	No	No	Unclear	Yes
Porzelius et al. [52]	Yes	Yes	No	No	No	Yes	No	Unclear	Yes
Puglisi et al. [40]	Yes	Yes	Yes	Not applicable	Not applicable	Yes	No	Unclear	Yes
Raymond et al. [57]	Yes	Yes	Yes	No	No	Yes	No	Unclear	Yes
Sallet et al. [41]	Yes	Yes	Yes	Not applicable	Not applicable	Yes	No	Unclear	Yes
Susmallian et al. [42]	Yes	Yes	Yes	Not applicable	Not applicable	Yes	No	Unclear	Yes
Telch et al. [58]	Yes	Yes	No data	Not applicable	Not applicable	Yes	No	Unclear	Yes
Tseng et al. [64]	Yes	Yes	No	Not applicable	Not applicable	Yes	No	Unclear	Yes
Wadden et al. [62]	Yes	Yes	No	Not applicable	Not applicable	Yes	No	Unclear	Yes
Wadden et al. [37]	Yes	Yes	No	Not applicable	Not applicable	Yes	No	Unclear	Yes
Yanovski et al. [65]	Yes	Yes	No	Not applicable	Not applicable	Yes	No	Unclear	Yes
De Zwaan [46]	Yes	Yes	Yes	Not applicable	Not applicable	Yes	No	Unclear	Yes

### Results of syntheses

Seventeen out of the 34 included studies reported data that was suitable for a meta-analysis, and we conducted 6 meta-analyses in total. The remaining 17 studies did not report data in a format that was suitable for a meta-analysis and were assessed qualitatively.

### Overall weight change in people with or without binge eating who received any type of weight loss treatments

**Fig. 2:**
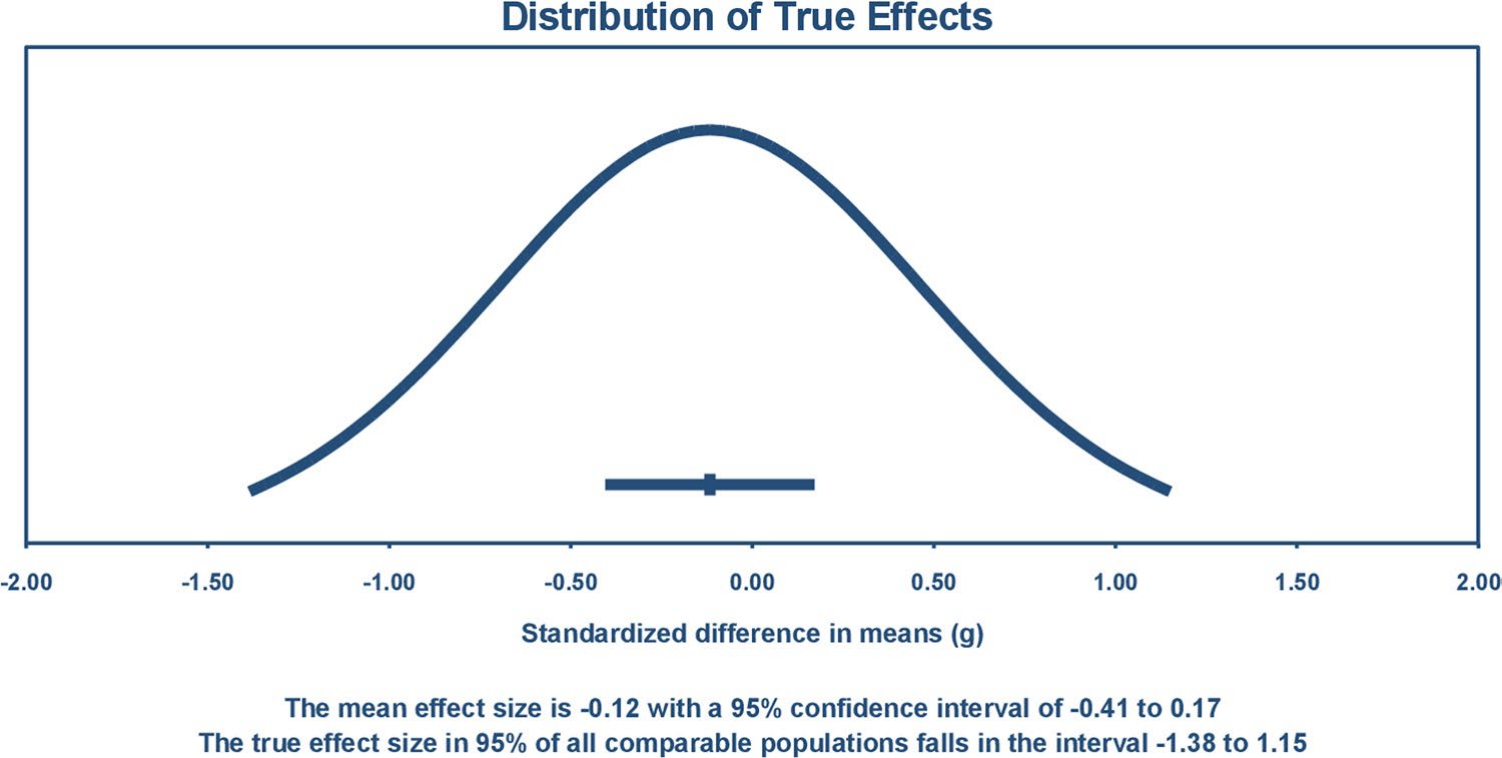
Distribution of true effects

One of our meta-analyses compared weight change in people with or without binge eating who received any type of weight loss treatment. This meta-analysis included the outcome of 21 weight loss treatments, that were reported in 17 studies, with a total of 3017 participants. This meta-analysis showed no significant difference in weight loss between people with or without binge eating, with an overall effect size (standardized mean difference, SMD) of − 0.117 (95% confident interval [CI] 0–0.405 to 0.171; *P* = 0.426), which is considered small by Cohen’s [66] definition. The studies in this meta-analysis showed heterogeneity. Heterogeneity was calculated by Tau^2^ (variance of true effect size) with a value of 0.345, and Tau (standard deviation of the true effect sizes) was 0.588 which is considered high, and *I*^2^ of 82.6. The prediction interval (PI) was − 1.38 to 1.15 (Fig. 2), which means that in 95% of all populations the true effect size would fall in this range.

Our subgroup analysis showed no statistically significant differences in body weight between people with or without binge eating at short (< 12 months) versus long (≥ 12 months) follow-up assessments following any type of weight loss treatment, with an overall effect size of − 0.118 (CI of − 0.419 to 0.184; *P* = 0.44). However, we found significant heterogeneity (see Table 3).

**Table 3:** Summary effect sizes and heterogeneity from the meta-analyses conducted in this systematic review

Category	No of studies	Summary effect		Heterogeneity				Prediction interval
		Size (95% confidence interval)	*P* value^a^	Q (degrees of freedom)	*P* value for *X*^2^ test on *Q*^b^	*I*^2^ (%)^c^	*Tau*^d^	
Overall studies	21	− 0.117 (− 0.405 to 0.426)	**0.042**	114.651 (20)	**0.000**	82.556	0.345	− 1.38 to 1.15
Intervention types								
Behavioral and/or nutritional interventions	11	− 0.084 (− 0.478 to 0.310)	0.675	10.052 (10)	0.436	0.521	0.000	
Bariatric surgery	6	− 0.538 (− 1.066 to − 0.010)	**0.046**	82.635 (5)	**0.000**	93.949	1.138	
Pharmacotherapy isolated or combined with behavior interventions	4	0.434 (− 0.216 to 0.184)	0.191	2.427 (3)	0.489	0.000	0.000	
Follow-up time								
Interventions with short follow-up (< 12 months)	13	− 0.202 (− 0.584 to 0.179)	0.299	97.914	**0.000**	87.744	0.676	
Interventions with long follow-up (≥ 12 months)	8	0.022 (− 0.468 to 0.513)	0.929	16.703	**0.019**	58.092	0.081	

^a^Summary effects are based on standard mean difference (SMD). Bold *P* values show statistically significant values (i.e., < 0.05)

^b^*P* value for *Q* statistics shows the result of testing the null hypothesis that all studies have the same underlying effect size, and the *Q* value is a measure of how likely the observed variations would be if that hypothesis were true

^c^I2 values show the observed heterogeneity in the meta-analysis is due to true variance i.e., sampling error

^d^Tau2 shows the estimations in the variance of the true effect sizes across different studies

### Assessment of weight change in people with or without binge eating in specific types of weight loss treatments

**Fig. 3:**
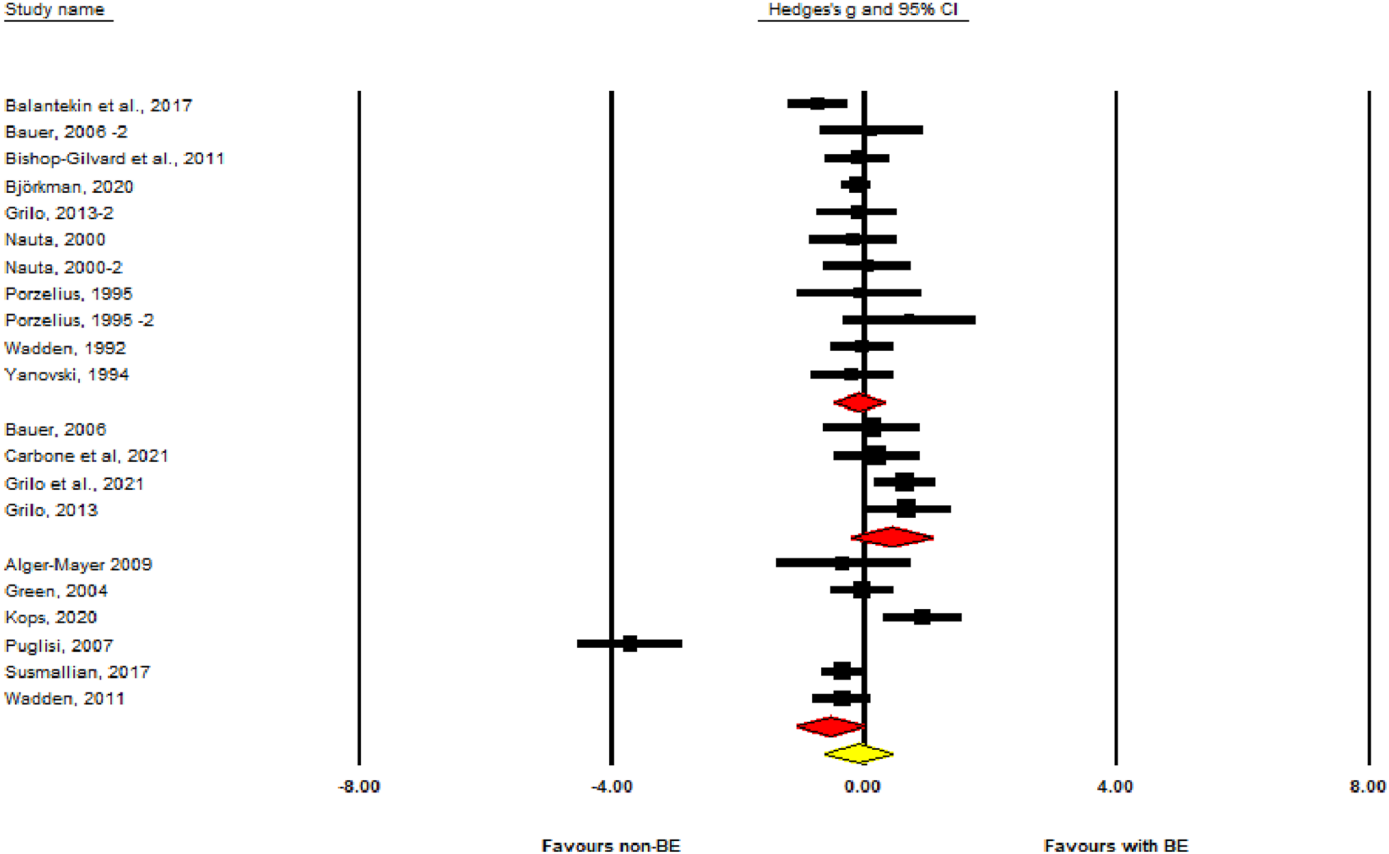
Forest plot by intervention subgroup and general. The first diamond (from the top-down) refers to behavioral and/or nutritional interventions, the second diamond refers to pharmacotherapy isolated or combined with behavior interventions, and the third diamond refers to weight loss surgery. The last diamond refers to all treatments combined

We also examined changes in body weight in people with or without binge eating in three sub-group analysis in our meta-analyses categorized by the type of weight loss treatment (i.e., bariatric surgery; pharmacotherapy isolated or combined with behavioral interventions; behavioral and/or nutritional interventions) (Fig. 3). We assumed a common among-study variance component across subgroups (pool within-group estimates of tau-squared) and combined the subgroups using random effects to yield an overall effect. The results of our meta-analyses showed that people without binge eating prior to bariatric surgery lost significantly more body weight compared to those with binge eating prior to bariatric surgery (SMD = − 0.538; CI = − 1.066 to − 0.010; *P* = 0.046). However, significant heterogeneity was observed between the studies (*P* value = 0.000), with a prediction interval (PI) from − 1.87 to 0.79. This precludes us from making a definitive conclusion regarding the superiority of bariatric surgery induced greater weight loss in people without binge eating compared to those with pre-treatment binge eating. When investigating the effect of pharmacotherapy isolated or combined with behavior interventions on weight loss, our results showed no difference in weight loss in people with or without pre-treatment binge eating (SMD = − 0.434; CI = − 0.216 to 1.084; *P* = 0.191). We did not find evidence of heterogeneity in this meta-analysis. Finally, our meta-analysis investigating the effect of behavioral and/or nutritional interventions on weight loss found no difference in weight loss in people with or without pre-treatment binge eating (SMD = − 0.084; CI = − 0.478 to − 0.310; *P* = 0.675). We did not find evidence of heterogeneity in this meta-analysis. In addition, our assessment of reporting bias assessment did not show any effect on our results.

## Studies that were not included in a meta-analysis, because data format was not suitable for a meta-analysis

### Weight loss in people with or without binge eating who underwent weight loss surgery

Six studies assessed weight loss in people with or without pre-treatment binge eating who underwent bariatric surgery. Two of these studies found that people with binge eating lost significantly less weight in comparison with those without binge eating [33, 41] and four of these studies did not find significant differences in weight loss in people with or without binge eating [34, 36, 38, 43].

### Weight loss in people with or without binge eating who received pharmacotherapy isolated or combined with behavior interventions

Three studies [44, 46, 47] assessed weight loss in people with or without pre-treatment binge eating who received pharmacotherapy isolated or combined with behavioral interventions. These studies did not find significant differences in weight loss in people with or without binge eating [44, 46, 47].

### Weight loss in people with or without binge eating who received behavioral and/or nutritional interventions

Eight studies [51, 56–58, 60, 61, 63, 64] assessed weight loss in people with or without pre-treatment binge eating who received behavioral and/or nutritional interventions. Two of these studies found that people with binge eating lost significantly less weight in comparison with those without binge eating [60, 61], and 1 study showed that people with binge eating lost significantly more weight than people without binge eating [63]. However, most studies (i.e., 5 out of 8) did not find significant differences in weight loss between people with or without pre-treatment binge eating who received behavioral and/or nutritional interventions [51, 56–58, 64].

## Discussion

The aim of this systematic review with meta-analyses was to compare changes in body weight in people with or without pre-treatment binge eating who received a variety of weight loss treatments. Overall, the meta-analyses found no differences in weight loss—between people with or without pre-treatment binge eating—at the end of treatment or last follow-up assessment reported in each study. There were also no differences in weight loss observed in sub-group analyses for different types of weight loss treatments or follow-up periods. While we found a difference in weight loss between people with or without pre-treatment binge eating in the studies of bariatric surgery, we were unable to conclude that there is a difference in weight loss due to high level of heterogeneity present. Furthermore, our qualitative analysis of studies that were not included in a meta-analysis (i.e., studies that did not report data in a format that was suitable for a meta-analysis) also showed no difference in weight loss in people with or without pre-treatment binge eating.

Our findings are in line with the results of a previous systematic review with meta-analysis that found no difference in weight loss between people with or without binge eating who underwent bariatric surgery [21]. In addition, our systematic review with meta-analyses expands the knowledge in this field as we found no difference in weight loss between people with or without binge eating who received other weight loss treatments (i.e., pharmacotherapy isolated or combined with behavior interventions; behavioral and/or nutritional interventions). Our findings contrasted with the findings of a matched-study meta-analysis that showed that people with pre-treatment BED lost significantly less weight compared to people without pre-treatment BED in a variety of weight loss treatments [26]. However, the method of that matched-study meta-analysis differs from our systematic review with meta-analyses [26]. For instance, as we mentioned in the Introduction, that matched-study meta-analysis did not comprehensively search the literature for all published studies that examined the effects of weight loss treatments in people with or without binge eating [26]. Moreover, that matched-study meta-analysis included only studies that were published until 2004, while our systematic review with meta-analyses included studies that were published until 2022 [26].

A potential explanation for our finding of similar weight loss in people with or without pre-treatment binge eating is that some weight loss treatments can potentially induce greater control over eating behavior. For example, some studies included in our systematic review showed that binge eating reduced once people received weight loss treatments [33, 37, 43–45, 47, 50, 52, 53, 55, 57, 60, 64]. Similarly, a randomized trial that was not included in our review showed that people with binge eating who received a dietary intervention reduced binge eating, uncontrolled eating, and emotional eating [67]. In addition, a longitudinal study showed that loss of control over eating and BED reduced 1 year after bariatric surgery (albeit loss of control over eating and BED resumed in the following years) [68]. Finally, a systematic review with meta-analysis found reductions in eating disorder symptoms, binge eating severity, and binge eating episodes in people with overweight or obesity who received behavior weight management interventions [69]. Overall, these studies suggest that weight loss interventions conducted under clinical supervision can potentially assist people in reducing binge eating at least during the treatment phase.

In conclusion, our systematic review with meta-analyses found no difference in weight loss in people with or without binge eating who received a variety of weight loss treatments. Thus, it is unlikely that pre-treatment binge eating will impede weight loss outcomes in people with overweight or obesity who received clinically supervised weight loss interventions. Notwithstanding that people with or without binge eating can reduce body weight similarly, psychological assessments of people with obesity seeking weight loss treatments can be useful to identify those who may benefit from eating disorder therapies.

### Strengths and limits

Our systematic review with meta-analyses has several strengths and limitations. A notable strength is that [28]. Our review was comprehensive, as it included varied types of weight loss treatments or combinations of treatments, and samples of males and females of different age groups. We also performed sub-group analyses (e.g., based on different treatment categories, or length of the last assessment) and conducted a qualitative analysis of studies that were not included in a meta-analysis, allowing us to gain a comprehensive understanding of weight change in individuals with or without pre-treatment binge eating who underwent various weight loss interventions. Our systematic review with meta-analyses was limited in that the risk of bias assessment showed that some of the included studies had a low-quality regarding method of randomization, allocation concealment, blind participants and assessors, and sample power calculation, which lowers the strength of data reported in these studies. In addition, this review was limited in that there were differences in the methods used to classify the occurrence of binge eating in the included studies. Some of the studies included participants that met full criteria for BED, while other studies included participants with subthreshold BED, or participants only with loss of control over eating. This is relevant as more extreme comparisons (e.g., people with BED versus people without binge eating) can potentially show significantly greater differences in weight loss [26]. Second, we did not examine the effects of weight loss treatments on binge eating behaviors. This is an important consideration, because the occurrence or absence of binge eating episodes during a weight loss treatment can potentially interfere with weight loss outcomes. For example, a previous study found that people with pre-treatment binge eating that stopped binge eating once they received an intensive lifetime intervention were just as successful to lose weight as people without binge eating [20]. In addition, most studies included in our systematic review did not assess whether participants engaged in other eating disorder behaviors (e.g., compulsive exercise, self-induced vomiting, or abuse of laxatives/diuretics) that can potentially influence weight loss outcomes. It is noteworthy that we did not investigate the safety of weight loss treatments on mental health of people with overweight or obesity and recurrent binge eating and the risk of transitioning to a restrictive eating disorder, such as atypical anorexia [70]. Even though behavior weight management interventions do not increase eating disorder behaviors in most adults, a small proportion of people (0–6.5%) can be at risk of experiencing eating disorder symptoms during or after behavior weight loss treatments [69]. Thus, in any event eating disorder therapies following updated guidelines should be provided to people with a high body weight and comorbid binge eating seeking weight loss treatments [71]. Finally, it should be noted that the differential weight loss in people with or without binge eating derives mostly from sub-analyses of the studies included in our review.

### What is already known about this subject?

Previous studies showed contrasting findings on whether pre-treatment binge eating can hinder weight loss in people who received obesity treatments.

### What do we now know as a result of this study that we did not know before?

Our systematic review with meta-analyses found no difference in weight loss in people with or without pre-treatment binge eating who received varied types of weight loss treatments. Weight loss treatments should not be withheld on the basis that they will not be effective in people with pre-treatment binge eating. However, further research is needed to investigate the safety and long-term impacts of weight loss treatments in people with recurrent binge eating.

## Data Availability

Not applicable.
